# The role of the Strait of Gibraltar in shaping the genetic structure of the Mediterranean Grenadier, *Coryphaenoides mediterraneus*, between the Atlantic and Mediterranean Sea

**DOI:** 10.1371/journal.pone.0174988

**Published:** 2017-05-01

**Authors:** Diana Catarino, Sergio Stefanni, Per Erik Jorde, Gui M. Menezes, Joan B. Company, Francis Neat, Halvor Knutsen

**Affiliations:** 1 MARE – Marine and Environmental Sciences Centre, University of the Azores, Department of Oceanography and Fisheries, Horta, Azores, Portugal; 2 Department of Oceanography and Fisheries, University of the Azores, Horta, Azores, Portugal; 3 Stazione Zoologica A. Dohrn, Villa Comunale, Napoli, Italy; 4 Centre for Ecological and Evolutionary Synthesis (CEES), Department of Biosciences, University of Oslo, Oslo, Norway; 5 Institut de Ciències del Mar (ICM-CSIC), Barcelona, Spain; 6 Marine Scotland-Science, Marine Laboratory, Aberdeen, United Kingdom; 7 Institute of Marine Research, Flødevigen, Norway; 8 Centre for Coastal Research, University of Agder, Kristiansand, Norway; National Cheng Kung University, TAIWAN

## Abstract

Population genetic studies of species inhabiting the deepest parts of the oceans are still scarce and only until recently we started to understand how oceanographic processes and topography affect dispersal and gene flow patterns. The aim of this study was to investigate the spatial population genetic structure of the bathyal bony fish *Coryphaenoides mediterraneus*, with a focus on the Atlantic–Mediterranean transition. We used nine nuclear microsatellites and the mitochondrial *cytochrome c oxidase I* gene from 6 different sampling areas. No population genetic structure was found within Mediterranean with both marker types (mean *Φ*_ST_ = 0.0960, *F*_ST_ = -0.0003, for both *P* > 0.05). However, within the Atlantic a contrasting pattern of genetic structure was found for the mtDNA and nuclear markers (mean *Φ*_ST_ = 0.2479, *P* < 0.001; *F*_ST_ = -0.0001, *P* > 0.05). When comparing samples from Atlantic and Mediterranean they exhibited high and significant levels of genetic divergence (mean *Φ*_ST_ = 0.7171, *F*_ST_ = 0.0245, for both *P* < 0.001) regardless the genetic marker used. Furthermore, no shared haplotypes were found between Atlantic and Mediterranean populations. These results suggest very limited genetic exchange between Atlantic and Mediterranean populations of *C*. *mediterraneus*, likely due to the shallow bathymetry of the Strait of Gibraltar acting as a barrier to gene flow. This physical barrier not only prevents the direct interactions between the deep-living adults, but also must prevent interchange of pelagic early life stages between the two basins. According to Bayesian simulations it is likely that Atlantic and Mediterranean populations of *C*. *mediterraneus* were separated during the late Pleistocene, which is congruent with results for other deep-sea fish from the same region.

## Introduction

Population genetic studies of species inhabiting the deepest parts of the oceans are still scarce compared to shallow or coastal water relatives. The deep-sea is historically described as a stable and homogeneous environment where the existence of barriers to dispersal and gene flow among populations are less evident [1]. Some studies support this pattern since genetic homogeneity over large spatial scales of several deep-sea species seems to prevail, for example the wreckfish (*Polyprion americanus* [2]), the hydrothermal vent shrimp (*Rimicaris exoculata*, [3]), the black scabbardfish (*Aphanopus carbo*, [4]), the blue hake (*Antimora rostrata*, [5]) and the orange roughy (*Hoplostethus atlanticus*, [6]). Nevertheless, other species such as roundnose grenadier (*Coryphaenoides rupestris*, [7]) and the bluemouth rockfish (*Helicolenus dactylopterus*, [8]) show a contrasting genetic pattern exhibiting significant levels of genetic divergence across their range. The mixed scenario of genetic patterns across deep-sea species is likely to reflect depth niche, bathyal heterogeneity and ocean currents, as well as differences among species in life histories and dispersal capabilities.

Although much work is still needed to understand the most important processes and mechanisms affecting dispersal and gene flow patterns in the deep sea, the number of studies relating oceanographic processes and topography with genetic patterns has been increasing in the last years (i.e. [9, 10, 11]). For some species, topographic features may act as physical barriers to the individual movements or by diverging water circulation, influencing both dispersal and gene flow. The Strait of Gibraltar, the shallow ridge that separates the Mediterranean basin from the Atlantic basins, has been brought to attention as an important phylogeographic barrier (reviewed by [12]). This strait is approximately 12.9 km wide and 284 m deep. At the top, the Modified Atlantic Water enters the Mediterranean while at deeper layers, the Intermediate Mediterranean Water (denser and saltier) flows toward the Atlantic [13].

Within the Mediterranean Sea, the Straits of Sicily and Messina may function as barriers between the western and the eastern Mediterranean which may influence gene flow of several marine organisms [14–17]. The Strait of Sicily is located between the island of Sicily and Tunisia and has approximately 360–430 m depth. The strait of Messina, located between the eastern tip of Sicily Island and mainland Italy, is a very narrow sill shallower than the strait of Sicily (3.1 km wide and approximately 80 m deep at the narrowest part [18]). Concerning oceanographic processes, the Almeria-Oran front (AOF) found between Spain and Algeria is one of the most studied dynamic systems acting as a barrier to gene flow in the Mediterranean (reviewed by [12]). The AOF [19] is driven by different water densities and is located at the eastern end of the Alboran Sea extending down to 300 m deep. It can severely limit the mixing of the epipelagic eggs and larvae between the Atlantic and the Mediterranean.

Within the North Atlantic Ocean the most prominent topographical feature is the Mid-Atlantic Ridge (MAR) located between the Azores and Iceland, rising from abyssal depths to about 800 m below the sea surface. The MAR is interrupted by a major fracture zone, the Charlie–Gibbs Fracture Zone (CGFZ; located about 52°N) which is the deepest connection between the north-eastern and north-western Atlantic and influences the pathway of the North Atlantic Current circulation (NAC, [20]). Due to the sharp changes in bathymetry in the CGFZ area, the NAC breaks into several branches, one of which forms the Sub-Polar Front (SPF, [21]). There is evidence that the SPF acts as a biogeographic barrier for zooplankton, and early life stages of epi- and mesopelagic fish [22–24].

The Mediterranean grenadier, *Coryphaenoides mediterraneus* (Giglioli, 1893), is a benthopelagic deep-sea macrourid fish that inhabits continental slopes, ocean ridges and seamounts usually at depths between 1000 m and 3000 m [25, 26], although there are records down to 4300 m depth [27]. Despite its name, this species is present not only in the Mediterranean Sea (e.g. [28, 29]), but has a wide distribution in the North Atlantic from Iceland [30] to Mauritania [31], as well as in the Gulf of Mexico [32]. The species feeds mainly on small invertebrates [33], and different diets for Atlantic and Mediterranean specimens have been described [34, 35]. Although key life history traits for the species have never been investigated, some biologic information (size and age) may suggest potential segregation between Atlantic and Mediterranean populations. Atlantic specimens can grow to a maximum of 73 cm total length (TL; [30]), but adult fish from the Mediterranean attain smaller sizes [36]. The species has been aged by reading of the increments in the sagittal otoliths, but the different methodologies have not been validated. In the Atlantic the older specimens studied were estimated to be more than 20 years old (max. 27 years old; [37, 38]) while in the Mediterranean adults were estimated to be maximum 6 years old [39]. Not much is known about the reproduction of this species, besides being oviparous and releasing eggs in the water column which are likely to be passively dispersed by ocean currents [30]. Population mixing in oviparous fishes with external fertilization can occur through the dispersion of eggs and larvae by ocean currents (e.g. [40– 42]) and/or through the active movements of juveniles or adult migrants (i.e. [2, 4]). Given that the Strait of Gibraltar is far shallower than the species’ vertical distribution, restriction of direct gene exchange between Atlantic and Mediterranean populations may occur. Furthermore, the lack of information on key aspects of the life history of *C*. *mediterraneus*, such as on the species early life stages, and the uncertainty of how bathymetry and oceanography affects dispersal potential, greatly limits the knowledge on population connectivity across the species’ range. Therefore, the aim of this study was to characterize the spatial population genetic structure of the bathyal bony fish *C*. *mediterraneus*, with special emphasis on the transition between Atlantic and Mediterranean Sea.

## Material and methods

### Ethics statement

No specific permits were required for the described field studies, since all the specimens used came from scientific cruises only. All specimens from this deep sea species were already dead when arrived on board, since they were fished from great depths. *C*. *mediterraneus* is neither an endangered nor a protected species.

### Sampling and biological data

**Fig 1:**
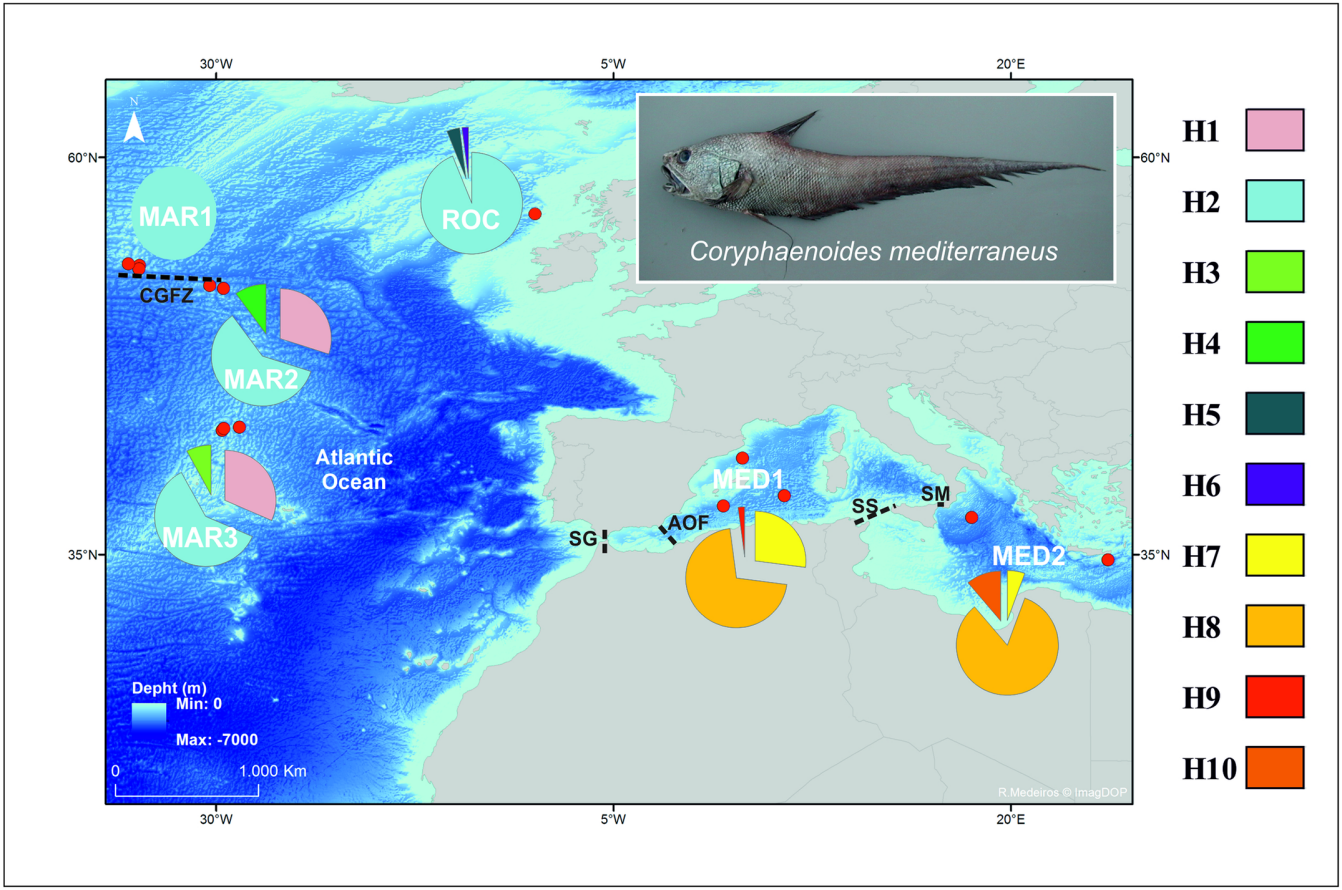
Map showing the sampling sites (red dots) of for *Coryphaenoides mediterraneus*. Pie charts report the haplotype distributions of the mtDNA COI, with the proportion of each haplotype within each collection site. Each haplotype is colour coded according to the legend on the right. Abbreviations of the collection sites are given in Table 1. Dark and dashed lines represent the potential barriers to gene flow: CGFZ—Charlie-Gibbs fraction zone; SG—Strait of Gibraltar; AOF- Almeria-Oran front; SS—Strait of Sicily; SM—Strait of Messina. Map under a CC BY license, with publish permission from R. Medeiros.

Specimens of *C*. *mediterraneus* were collected from 3 main geographic areas (Fig 1; Table 1): Mid-Atlantic Ridge (MAR), Rockall Trough (ROC) and Mediterranean Sea (MED). The Mid-Atlantic Ridge collection site was further divided in 3 sub-samples along the ridge (MAR1, MAR2 and MAR3) and the Rockall samples consisted in assemblages from two years (2011 and 2012). The Mediterranean samples include specimens collected in the Western Mediterranean (MED1, fish collected at three main sites; Table 1 and Table A in S1 Appendix) and in the Eastern Mediterranean (MED2, two collection sites; Table 1 and Table A in S1 Appendix). All samples were collected using deep-water bottom trawl nets during several scientific cruises [25, 43, 44]. Small portions of white muscle or gill tissue were collected for genetic analyses, and preserved in 95% ethanol until DNA extraction.

**Table 1:** Information on the *C*. *mediterraneus* collection sites.

Sampling area	Code	Coordinates		Capture depth (m)	SWBT (°C)
		Latitude	Longitude		
**Mid-Atlantic Ridge combined**	**MAR**				
Northern Mid-Atlantic Ridge	MAR1	53°08' N	34°46' W	2306–2374	2.99
	MAR1	52°58' N	34°52' W	1630–1670	3.13
	MAR1	53°16' N	35°31' W	2522–2567	3.08
Middle Mid-Atlantic Ridge	MAR2	51°45' N	29°33' W	1872–1950	3.43
	MAR2	51°55' N	30°25' W	1872–1959	3.42
Southern Mid-Atlantic Ridge	MAR3	42°48' N	29°38' W	2063–2107	3.93
	MAR3	42°55' N	29°32' W	1702–1767	5.01
	MAR3	43°01' N	28°33' W	2593–2607	3.79
**Rockall**	**ROC**				
Rockall 2011	ROC11	57° N	9°30' W	1250–1800	3.73–4.97
Rockall 2012	ROC12	57° N	9°30' W	1500–2012	3.37–4.01
**Mediterranean combined**	**MED**				
Western Mediterranean	MED1	41°03' N	3°05' E	1730–2230	13.00*
	MED1	38°02' N	1°54' E	2000	13.21
	MED1	38°41' N	5°41' E	2800–2850	13.32
Eastern Mediterranean	MED2	37°19' N	17°30' E	1915–3332	13.95
	MED2	34°38' N	26°05' E	2053–2966	13.91

Details on the geographic coordinates, the interval of depth of capture and sea water bottom temperature (SWBT) at each collection site. In bold are major sampled areas.

* This value is approximate and was retrieved from the literature.

Length data (pre-anal fin length, PAFL) was collected from a total of 152 specimens in MAR, 90 in ROC and 121 from MED. The MED data includes a set of 28 specimens collected in 2013, which were not included in the genetic screening.

### DNA extraction and amplification

#### Mitochondrial DNA (mtDNA)

Genomic DNA was extracted using the E.Z.N.A.^®^ (EaZy Nucleic Acid Isolation) Mag-Bind Tissue DNA kit (OMEGA bio-tek, USA) with the KingFisher mL magnetic particle processor (Thermo—Electron Coorporation, USA), according to the manufacturer’s instructions. The mitochondrial cytochrome *c* oxidase subunit I (COI) was partial amplified (613 bp) for 193 individuals by polymerase chain reaction (PCR) using the FishF2 and FishR2 primers [45]. PCR amplification were performed in reactions of 20 μL of volume containing 0,5 μL of each primer (10 μM), 1 μL of DNA template (10–20 ng), 10 μL of PCR Master Mix (Promega Corporation, USA), 8 μL of dH_2_O and were conducted under the following thermal cycle conditions: initial denaturation of 2 min at 94°C, followed by 35 cycles of denaturation for 30 s at 94°C, annealing for 60 s at 55°C, extension for 1 min 35 s at 72°C, with a final extension of 7 min at 72°C. No template controls were included to check for possible DNA contamination. Electrophoresis of PCR products was performed on a 1% agarose gel to assure the integrity of the products. Finally, all amplified products were purified using ExoSAP-IT (USB Corporation, USA) and sequenced commercially at BMR Genomics (Padua, Italy). All haplotypes were confirmed by sequencing both the forward and reverse strand for at least one individual per haplotype.

#### Nuclear DNA (microsatellites)

Genomic DNA was extracted using the E.Z.N.A.^®^ Tissue DNA Kit (OMEGA bio-tek) according to the manufacturer’s instructions. A total of 375 fish were genotyped for 9 nuclear microsatellite loci developed for other related species (Table B in S1 Appendix; described in [46–48]). After optimization of the PCR conditions, two primers (CaraA106a and Mbe03) were run as a duplex, four primers (CaraA10, CaraA102, CaraB1 and CaraC1) as a multiplex and all the others were run solo (Table B in S1 Appendix). Microsatellite amplification was performed in 10 μL reaction volumes, each containing 0.15–0.25 μL of the forward primer (10 μM) with a fluorescent label (VIC, PET, NED or 6-FAM), 0.15–0.25 μL of the reverse primer (10 μM), 1 μL of the DNA template (10–20 ng), 0.10–0.16 μL of *Taq* DNA Polymerase (5 U/ μL; Qiagen, Germany), 3 μL of dNTPs (1 mM), 1 μL of 10x PCR Buffer (Qiagen) and dH_2_O to reach the final volume. Thermal cycling conditions consisted in an initial denaturation of 4 min at 95°C, followed by 30–45 cycles of denaturation for 45 s at 95°C, 60 s at the corresponding annealing temperature (Table B in S1 Appendix), extension for 60 s at 72°C, with a final extension of 15 min at 72°C. PCR products were run for fragment analyses on an ABI Prism 3130*xl* (Life Technologies). All genotypes were scored independently by two persons using GeneMapper software (version 4.0; Life Technologies).

### Statistical analyses

#### mtDNA sequences

Four mtDNA COI sequences of *C*. *mediterraneus* (three from MAR and one from Greenland) were available from Barcode of Life Data Systems (BOLD; sample ID: ME-9911; ME-11972; ME-13727; GLF011), and were used for comparison in the haplotype network. All sequences were aligned using SEAVIEW [49] and CLUSTALX (version 1.8.3.; [50]). A haplotype network was constructed using the Median Joining Network (MJN) method [51] and the Maximum Parsimony approach [52], with Network software version 4.6.1.0. (fluxus-engineering.com) using the default parameters.

Genetic diversity indices, including number of haplotypes (*H*_*n*_), haplotype diversity (*H*_*d*_) and nucleotide diversity (*π*), were calculated for each location. Genetic distance (*Φ*_ST_) between localities was estimated based on the mean number of pairwise differences among sequences in Arlequin (version 3.5.1.2.; [53]). The neutrality tests of Tajima’s *D* [54] and Fu’s *F*_S_ [55] were conducted based on an infinite-site model without recombination in Arlequin. The analyses were run using 10000 permutations of the data. *P*-values were interpreted under the False Discovery Rate approach (FDR; [56]) in multiple testing situations. Ramos-Onsins & Rozas’s *R*_2_ [57] was determined using DnaSP (version 5.1; [58]).

A Principal Component Analyses (PCA) was performed to visualise the genetic structure in the dataset using Primer software (version 6; [59]) based on the COI haplotype frequencies.

In order to estimate the average evolutionary divergence over sequences, the MEGA software (version 6.0 [60]) was used to calculate the average *p*-distances between Atlantic and Mediterranean haplo-groups with 10000 bootstrap replications. Such analyses was also performed between *C*. *mediterraneus* and four of its closest relatives (*C*. *striaturus*, *C*. *murray*, *C*. *carapinus* and *C*. *brevibarbis;* GenBank accession numbers are KX656427.1, KX656428.1, KX656411.1, KX656410.1, KX656382.1, KX656381.1, KX656377.1, KX656376.1 and KX656375.1, respectively) according to mtDNA COI phylogenetic reconstruction [61], for comparisons purposes. For this analysis a new alignment was created and trimmed, at both 5’ and 3’ ends, to 598 bp in order to fit all sequences.

#### Microsatellites

Gene diversity in the total sample (*H*_T_ [62]), observed (*H*_O_) and expected (*H*_E_) heterozygosity, observed number of alleles and allelic richness (at each locus and for each sampling locality) was calculated using FSTAT (version 2.9.3 [63]) and diveRsity R package [64]. Deviations from the Hardy–Weinberg equilibrium (HWE) expectations were accessed by estimating genotype proportions within loci by means of the inbreeding coefficient *F*_IS_ [65], and tested using two-sided probability exact tests in the genepop software (version 4.6 [66]). The FDR approach was applied when interpreting the resulting *P*-values [67]. Micro-Checker software (version 2.2.3 [68]) was used to check the presence of null alleles. BayeScan [69] and Lositan [70] were used to look for potential selection signatures between Atlantic and Mediterranean. BayeScan was run using the default parameters (total of 100 000 interactions and 50 000 bun-in). Lositan was run using the recommended options (on for ‘neutral’ mean *F*_ST_ and force mean *F*_ST_), the stepwise- mutation model (SMM) and 50 000 simulations.

Genetic differences among localities were quantified by *F*_ST_ estimator [65], over all sampling localities and for pairs of samples. Statistical significance of pairwise *F*_ST_ tests was assessed by *G*-test for allele frequency differences in genepop using 10000 dememorizations and batches, and 10000 iterations per batch. *F*_ST_ was also estimated with Arlequin based on the pairwise differences using 10000 permutations of the data. *P*-values were evaluated for significance under the FDR approach in multiple tests situations [56]. The genetic structure for the temporal replicates at ROC were quantified by *F*_ST_ and tested for genetic divergence as described above, before pooling them together.

Genetic differentiation patterns among samples were visualized by applying a PCA on allele frequencies using PCA-GEN (version 1.2.1[71]) and significance in each axis was tested using 10000 data randomizations

The Bottleneck software (version 1.2.02 [72]) was applied to investigate population declines, using the SMM and TPM model, the later with 70–90% stepwise mutations and 10–30 variance, and based on 10000 interactions. The Wilcoxon test was used to check for significant heterozygosity excess.

#### Combined markers

Statistical power of the obtained statistically significant results under different true levels of *Φ*_ST_ and *F*_ST_ tests was estimated using POWSIM (version 4.1 [73]), adjusting the number of generations of drift (*t*) with an effective population size (*N*_*e*_) of 3000 to yield the desired level of divergence. This *N*_e_ value, was chosen to be above the recommended minimum of 2000 (POWSIM manual), to minimize loss of alleles and its negative effect on power estimates. Both markers were tested independently. The percentage of significant outcomes (*P* < 0.05) is interpreted as the power of the data to detect the defined level of genetic divergence. A sampling scheme corresponding to the empirical data in each marker was used and the analyses were conducted using 1000 dememorizations, 100 batches and 1000 interactions per batch. Genetic isolation-by-distance (IBD) within the Atlantic was tested for both markers using ISOLDE as implemented in genepop, using 10000 permutations of the data for the Mantel tests. *F*_ST_ and *Φ*_ST_ semi matrices were calculated as specified above and geographic distances were calculated as the shortest possible oceanic path between sampled locations using Google Earth (Google Inc.).

Jost’s D statistics [74] was calculated in addition to *F*_ST_ and *Φ*_ST_ for comparison purposes. Pairwise *D*_est_ values were estimated in GeneALEx [75], using 9999 permutations. *P*-values in multiple tests situations were evaluated for significance using the FDR approach.

Population structure was characterized by the hierarchical analysis of molecular variance (AMOVA) using ARLEQUIN based on pairwise differences and 10000 permutations. Multiple groupings of samples were tested, based initially on geographical location (e.g. Atlantic and Mediterranean) and secondly based on PCA and pairwise *Φ*_ST_ results. The optimal grouping was selected based on largest *F*_CT_ (variance between groups/ regions) in relation to *F*_SC_ (variance between populations within groups/regions) values.

Spatial population genetic structure was also investigated by the Bayesian clustering algorithm implemented in Geneland (version 4.0.3 [76]) running under the R statistical environment [77]. Since the presence of null alleles may affect clustering analyses, we used Geneland´s option for overcoming this problem by using an additional computing step, in which genotype ambiguity (homozygotes) are accounted for and null alleles frequencies are estimated along the clustering algorithm. Two runs were performed (microsatellites): first the dataset was analysed based on the individual genotypes of the nine microsatellite loci using the null alleles option; a second run was performed by removing the loci displaying potential null alleles (Crup7 and CaraA10). A separated run was performed for mtDNA COI. Each run consisted of 2500000 interactions, 5000 burn-in and a thinning of 100. The number of genetic populations was set to 6 (accordingly to sampling areas) with correlated allele frequencies and spatial model. The number of clusters was inferred from the modal value of K with the highest posterior probability. To obtain a map of population membership and *F*_ST_ values between the clusters, the study area was divided into 10000 rectangles (100 by 100, equivalent to approximate 1250 km^2^).

Approximate Bayesian Computation (ABC, [78]), implemented in DIYABC (version 2.0 [79]), was used to evaluate the time since population split (*t*) between Atlantic and Mediterranean and long-term effective population size (*N*_e_) of both populations and for both types of markers. Because DIYABC requires the same number of end populations in all competing scenarios, it is not possible to compare directly scenarios that account for the different genetic structure found in the Atlantic with the different marker types (see Results section). Therefore, a subset of samples (ATL = MAR2+MAR3, MED) was used instead of the complete dataset of *C*. *mediterraneus* in order to perform computations using both maker types in the same analyses. A total of four different scenarios were tested (Fig A in S1 Appendix): *Scenario 1* –constant effective population size for ATL and MED after population split; *Scenario 2* –MED population size changes after population split while ATL population size remains constant over time; *Scenario 3* –ATL population size changes after population split while MED population size remains constant over time; *Scenario 4* –both ATL and MED population sizes change after population split. A total of 6000000 datasets were simulated to build the reference table. Several run trials were performed in order to choose the range of values that best fitted the observed data [80] and the final priors intervals were set as follows: *N*_ATL_ = 10–2000000; *N*_MED_ = 10–500000; *t* = 1–70000; *t1* = 1–20000, *t1*<*t*; *Nb*_ATL_ = 1–40000; *Nb*_MED_ = 1–40000. The mean mutation rate was set between 1.0 x 10^−6^ and 5.0 x 10^−4^ for the 9 microsatellites markers leaving the other parameters as default. For mtDNA COI, the most appropriate nucleotide substitution model was selected from the hierarchical series of likelihood ratio test, implemented in MEGA. The Kimura 2-parameter model (I = 0, G = 0) [81] had the lowest Bayesian Information Criterion (BIC [82]) value and was therefore selected. The mutation rate for COI was set between 1.0 x 10^−9^ and 8.0 x 10^−8^ while all other parameters were left as default. The summary statistics used in the analyses (Table C in S1 Appendix) selected according to the recommended by Cornuet [80, 83]. The posterior probability of each scenario was estimated using logistic regression [80, 83] on the 1% of simulated datasets closest to the observed dataset and 10 intermediated values. For the chosen scenario, type errors I (probability of being the true model, but not selected) and II (probability of not being the true model, but it is selected) were estimated computing 500 datasets for each competing scenarios. The posterior distribution of the parameters was estimated by the *logit* transformation and using 10000 selected data. Finally, a “model check” was performed using all the other summary statistics not used for building the reference table [80] (Table C in S1 Appendix). The discrepancy between the model and the observed data was assessed by a PCA using 10000 simulated datasets. Time of divergence in years was estimated assuming a generation time of 9 years, data retrieved from FishBase [30].

## Results

### Genetic variability

For the mtDNA, 193 COI sequences (613 bp) were amplified containing a total of 11 polymorphic sites (9 transitions, 2 transversions). From the resulting 10 haplotypes, 3 were singletons. The estimated haplotype diversity was low for the three main geographic regions, varying between 0.119 and 0.409, whereas the nucleotide diversity ranged from 0.0002 to 0.0009, for ROC and MED, respectively (Table 2). Considering only MAR sites, despite the approximately same number of individuals analysed in the north (MAR1, n = 30) and in the south (MAR3, n = 38) the haplotype diversity was much lower in the north (*H*_n_ = 0.000, as only the H2 haplotype was observed at MAR1, see Fig 1) than in the south (*H*_n_ = 0.542; Table 2). Estimates of evolutionary divergence over sequence pairs between groups are presented in Table D in S1 Appendix. Sequences divergence between the two *C*. *mediterraneus* haplo-groups (Atlantic and Mediterranean) was on average of 0.54% ± 0.20% (± SE), and the average *p*-distances between *C*. *mediterraneus* and other closely related *Coryphaenoides* species varied between 5.74% ± 0.95% (*C*. *striaturus*) and 11.29% ± 1.31% (*C*. *brevibarbis*).

**Table 2:** List of sampling sites with number of *C*. *mediterraneus* specimens collected for genetic analyses (*N*), and genetic diversity indices for the microsatellites and mtDNA COI markers.

Sampling area	Code	Nuclear microsatellites							Mitochondrial COI			
		N	*H*_o_	*H*_E_	*F*_IS_^1^	*F*_IS_^2^	A	*R*_s_	N	*H*_n_	*H*_d_	π
**Mid-Atlantic Ridge combined**	**MAR**	146	0.42	0.43	0.035	-0.014	10.3 (15)	9.4	78	4 (3)	0.394	0.0008
Northern Mid-Atlantic Ridge	MAR1	61	0.41	0.42	0.035	0.003	8.7 (7)	4.8	30	1	0.000	0.0000
Middle Mid-Atlantic Ridge	MAR2	13	0.35	0.38	0.130	0.080	4.0	3.9	10	3 (1)	0.600	0.0011
Southern Mid-Atlantic Ridge	MAR3	72	0.44	0.45	0.017	-0.044	8.7 (4)	4.7	38	3 (2)	0.542	0.0012
**Rockall**	**ROC**	132	0.43	0.43	0.003	-0.032	9.6 (12)	8.9	49	3 (2)	0.119	0.0002
Rockall 2011	ROC11	90	0.43	0.43	0.018	-0.011	9 (8)	4.6	49	3 (2)	0.119	0.0002
Rockall 2012	ROC12	42	0.45	0.44	-0.029	-0.072	6.4 (2)	4.6	—	—	—	—
**Mediterranean combined**	**MED**	97	0.36	0.38	0.048	0.012	7.2 (2)	7.2	66	4 (4)	0.409	0.0009
Western Mediterranean	MED1	78	0.35	0.37	0.068	0.043	6.4	4.1	48	3 (1)	0.434	0.0008
Eastern Mediterranean	MED2	19	0.41	0.39	-0.027	-0.100	4.9	4.2	18	3 (1)	0.307	0.0009

*H*_O_, observed heterozygosity; *H*_E_, expected heterozygosity; *F*_is_, inbreeding coefficient with ^**1**^ nine loci and ^**2**^ seven loci (removing Crup7 and CaraA10); no significant deviations from HWE were found across sampling localities; *A*, mean number of alleles (number of unique alleles for the sampled location); *R*_s_, mean allelic richness (minimum sample size of 95 individuals for general areas (MAR, ROC, MED) and 12 individuals for sub-areas); *H*_n_, number of haplotypes (unique haplotypes); *H*_d_, haplotype diversity; π, nucleotide diversity.

Regarding the microsatellites markers, a total of 375 fish were genotyped at 9 loci. No locus had more than 2% of missing data. The levels of observed (*H*_O_) and expected (*H*_E_) heterozygosity were generally low, varying between 0.35 (MAR2 and MED1) and 0.45 (ROC12) for the former, and between 0.37 (MED1) and 0.45 (MAR3) for the later. Allelic richness (*R*_s_) displayed a similar pattern for the three main sampled regions, with no statistical difference in the levels found inside the MED (7.2) compared to ROC (8.9; Mann-Whitney *U* = 32, *P*>0.05 two-tailed) and MAR (9.4; Mann-Whitney *U* = 35, *P*>0.05 two-tailed; Table 2). The overall genetic diversity in the total sample (*H*_T_) over the nine loci was low (0.422), however it varied among loci, from 0.021 (CaraA102) to 0.926 (Crup7). The total number of alleles per locus ranged from 2 (CaraA102) to 35 (Crup7; Table B in S1 Appendix). The two side probability test found deviations from the HWE for two of the nine loci (Crup7 and CaraA10) in the total sample, even after FDR correction (Table B in S1 Appendix). Furthermore, MicroChecker reported the possible existence of null alleles at these two loci. No loci showed evidence of being under directional selection either with BayeScan or with Lositan. However locus Crup7 was a candidate to balancing selection (α = -1.36, q-value = 0.03) in BayeScan.

### Spatial and temporal structure

#### mtDNA

**Fig 2:**
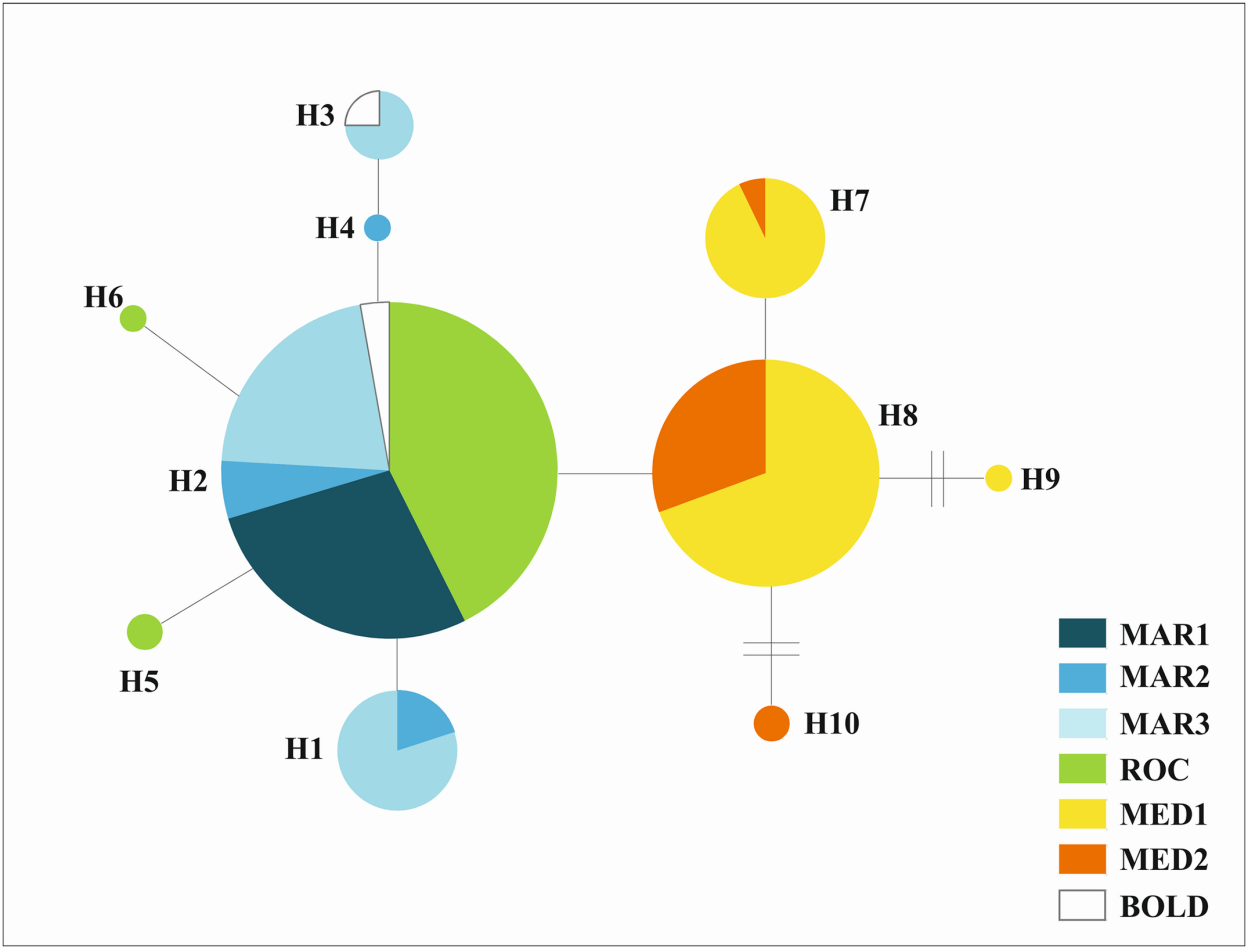
Median Joining Network from 613 bp mtDNA COI sequences. Size of circles (haplotypes) is proportional to the relative frequencies in the sample, and colour coded according to sampling locality; abbreviations are given in Table 1. Each branch indicates a single nucleotide substitution, except when noted. BOLD code represents the four sequences retrieved at Bold Systems for comparison (see Material and methods).

The spatial genetic patterns were illustrated with a haplotype network for the mtDNA COI (Fig 2), where no shared haplotypes were found between Atlantic and Mediterranean sites. The Atlantic and Mediterranean haplo-groups were separated by a single mutation step (a nucleotide transition A‹−›G) at position 91 in the alignment. The haplotype network consists of two main haplotypes occurring in high frequencies, one in the Atlantic (H2, 82% of the Atlantic samples) and the other in the Mediterranean (H8, 74% of the Mediterranean samples), and several other lower frequency derived haplotypes.

Very high and significant pairwise estimates of genetic differentiation between Atlantic and Mediterranean were found (mean *Φ*_ST_ = 0.7171, *P* < 0.001; mean *D*_est_ = 1.0000, *P* < 0.001; Table 3), even after FDR adjustment for multiple pairwise tests. Within the Mediterranean the *Φ*_ST_ pairwise comparisons between Western and Eastern basins came out non-significant (*Φ*_ST_ = 0.0960, *P* > 0.05; *D*_est_ = 0.0369, *P* > 0.05), after applying the FDR adjustment. Within the Atlantic Ocean, sampling sites exhibit a more complex genetic pattern with low levels of differentiation observed between MAR1 and ROC (*Φ*_ST =_ 0.0008, *P* > 0.05; *D*_est_ = 0.0014, *P* > 0.05), and also between MAR2 and MAR3 (*Φ*_ST =_ -0.0567, *P* > 0.05; *D*_est_ = 0.0000, *P* > 0.05). However, significant levels of genetic differentiation were found between the two northern (MAR1/ROC) and the two southern locations (MAR2/MAR3) (MAR1/ROC *vs*. MAR2/MAR3: *Φ*_ST_ = 0.1933 to 0.3617, *P* < 0.01; *D*_est_ = 0.1343 to 0.1686, *P* < 0.01; Table 3). The power analyses indicated that our minimum detection limit was a *Φ*_ST_ = 0.0643 with ≥ 95% confidence. Mantel tests did not support any significant relationship between *Φ*_ST_ and geographic distance (*a* = 0.2599, *b* = -6.9 × 10^−5^; *P* > 0.05) for the Atlantic samples.

**Table 3:** Pairwise comparisons and tests for differentiation between localities for microsatellites (below diagonals) and mtDNA COI (above diagonals). Upper table: pairwise *F*_ST_ and *Φ*_ST_; lower table Jost's D.

*F*_ST_*/ Φ*_*ST*_	MAR1	MAR2	MAR3	ROC	MED1	MED2
MAR1	—	0.3617**	0.1933***	0.0008	0.7798***	0.8389***
MAR2	-0.0021	—	-0.0567	0.3022**	0.6891***	0.6524***
MAR3	0.0025	-0.0011	—	0.2117***	0.6617***	0.6222***
ROC	0.0008	-0.0026	0.0022	—	0.7800***	0.8177***
MED1	0.0195***	0.0414***	0.0314***	0.0215***	—	0.096
MED2	0.0239*	0.0427**	0.0364***	0.0251***	-0.0003	—
**Jost’s *D***_***est***_						
MAR1	—	0.1558**	0.1686***	0.0014	1.0000***	1.0000***
MAR2	0.0000	—	0.0000	0.1343**	1.0000***	1.0000***
MAR3	0.0020	0.0000	—	0.1521***	1.0000***	1.0000***
ROC	0.0005	0.0000	0.0017	—	1.0000***	1.0000***
MED1	0.0132***	0.0256***	0.0288***	0.0154***	—	0.0369
MED2	0.0179***	0.0291**	0.0294***	0.0195***	0.0000	—

Significant values are in bold (significant at alpha = *0.05, ** 0.01, *** 0.001), after the FDR adjustment.

Sample site abbreviations are given in Table 2.

**Fig 3:**
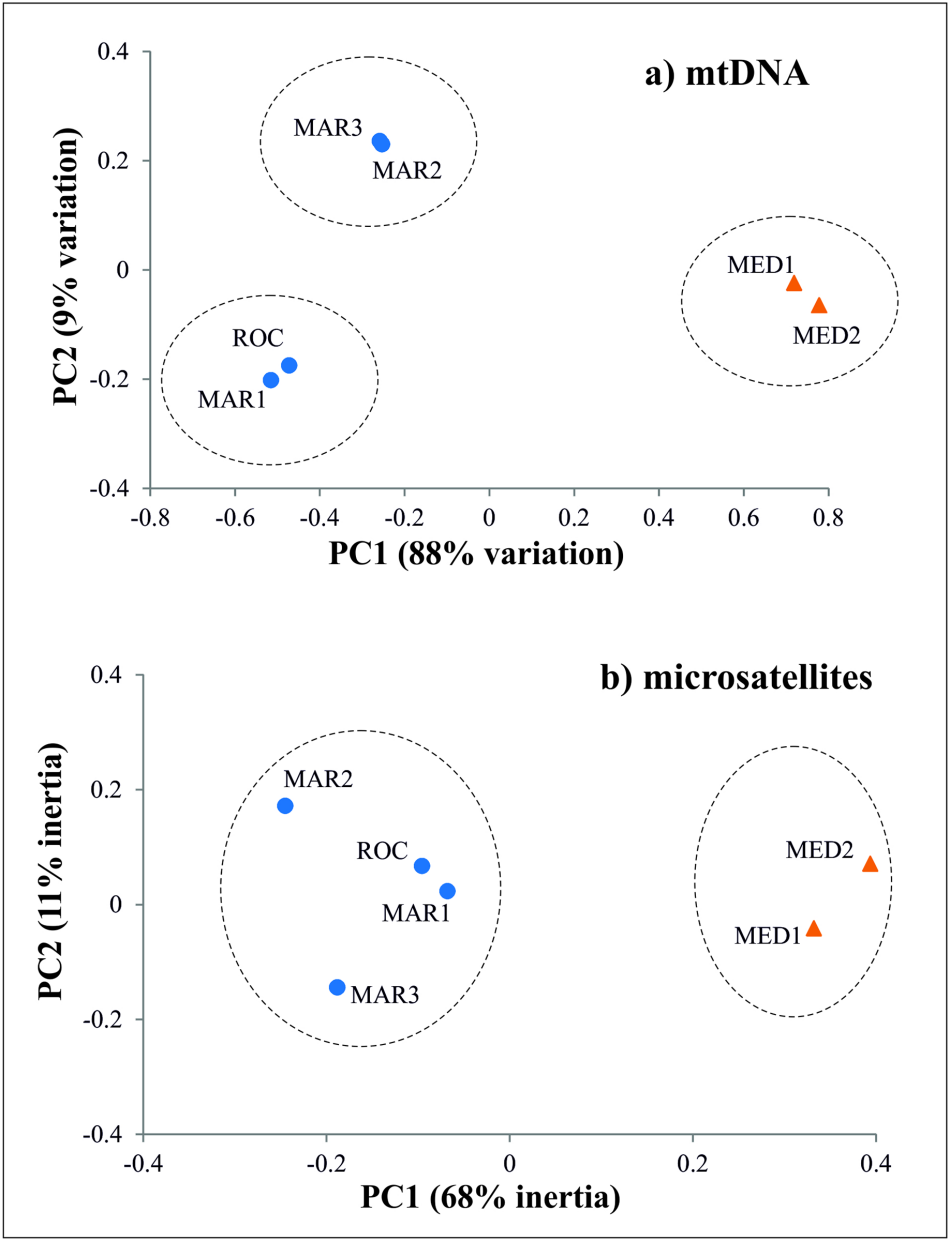
Principal Component Analyses (PCA). Analyses based on the haplotype frequencies of the mtDNA COI **(a)** and allele frequencies of the 9 microsatellite loci **(b)**. Triangles symbols refer to Mediterranean and dots to Atlantic localities. Dashed circles refer to grouping structure suggested by AMOVA and Geneland (mtDNA), and by PCA, AMOVA and Geneland analyses (microsatellites). Abbreviations are given in Table 1.

The PCA results (Fig 3a) show a separation between Atlantic and Mediterranean samples along the first axis which explains most of the variation (88%).

**Fig 4:**
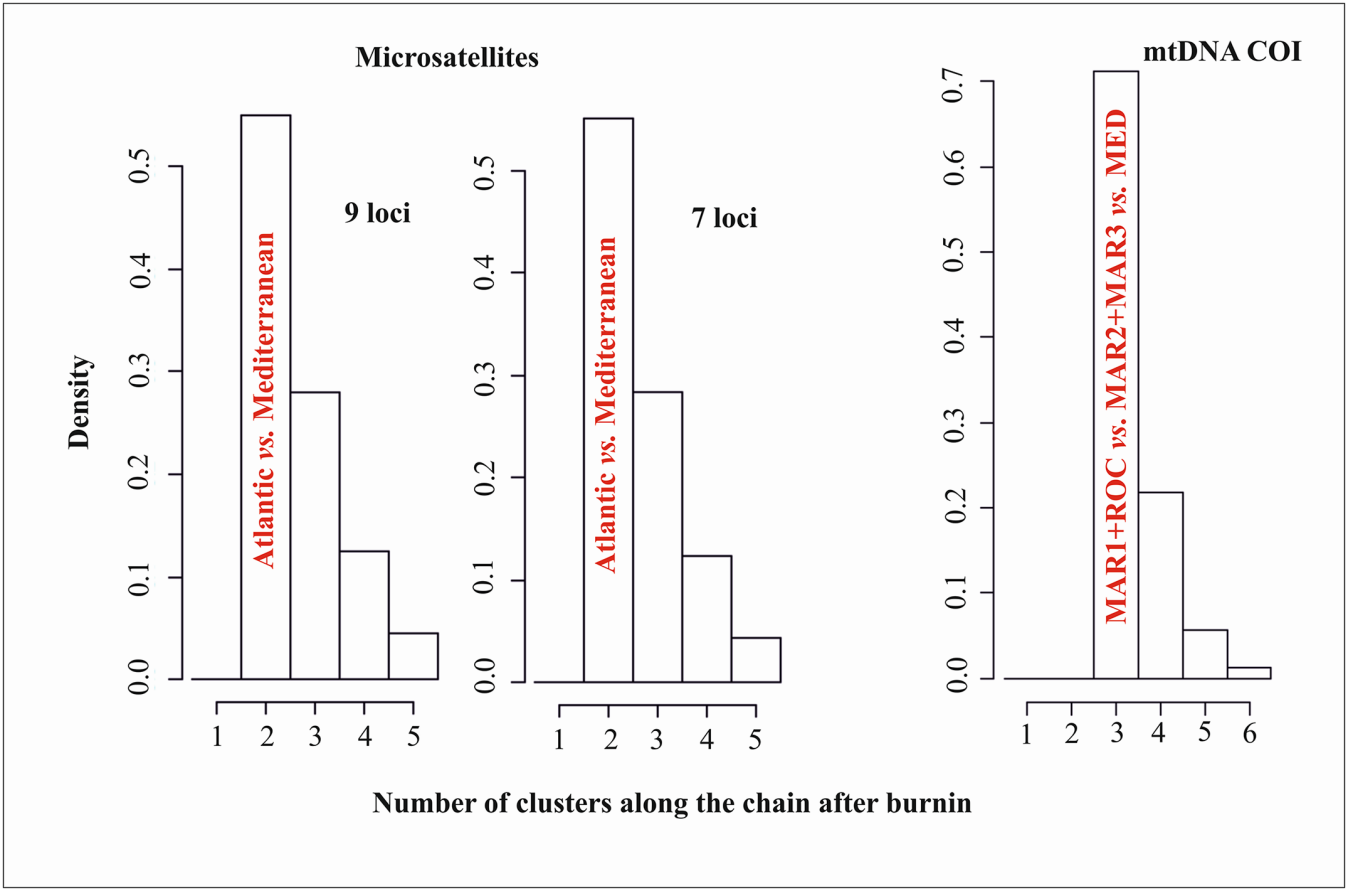
Posterior density distribution of the number of clusters estimated with Geneland. For nuclear microsatellites (on the left) it is shown both the results for 9 loci using the null alleles option, and using only the 7 loci without null alleles. On the right it is shown the estimated number of clusters for the mtDNA COI.

AMOVA was performed using multiple grouping criteria in order to investigate the optimal grouping for the dataset (Table E in S1 Appendix). The best grouping was (MAR1, ROC) (MAR2, MAR3) (MED1, MED2) since it explains the majority of the variance observed ‘among groups’ (i.e. the largest *F*_CT_), and minimizes the ‘within samples’ variation (*F*_SC_). Therefore the best population grouping suggests the separation of the Atlantic and Mediterranean populations. Within the Atlantic MAR1 and ROC are grouped together and are distinct of MAR2 and MAR3, which also are grouped together. In the same way, Geneland analyses revealed three clusters corresponding to MAR1/ROC, MAR2/MAR3 and MED populations (Fig 4).

#### Microsatellites

Significant genetic differentiation was found over all sampling localities and loci (*F*_ST_ = 0.0149, *P* < 0.001) even when removing the two loci showing HW deviations and possible presence of null alleles (*F*_ST_ = 0.0116, *P* < 0.001, Table B in S1 Appendix). However when looking to each locus separately three of the nine loci did not show significant genetic differentiation across sampling localities (CaraA109, CaraA10 and CaraA102, *P* > 0.05; Table B in S1 Appendix). No deviations from HWE were found across sampling localities (Table 2).

Regarding the temporal analysis for the ROC samples, no genetic heterogeneity was detected when comparing 2011 and 2012 samples (*F*_ST_: 2011 *vs*. 2012 = -0.0004, *P* >0.05), therefore the two samples were pooled together in one ROC sampling for further analyses.

The pairwise comparisons of both *F*_ST_ and *D*_est_ showed an identical genetic pattern across sampled localities (Table 3). *F*_ST_ calculations with both Genepop and Arlequin retrieved exactly the same genetic pattern across localities, after FDR approach (Table 3 and Table F in S1 Appendix). Results suggest the existence of a genetic break between Atlantic and Mediterranean since all comparisons resulted in high and significant values (overall *F*_ST_ = 0.0245, *P* < 0.001; *D*_est_ = 0.0180, *P* < 0.001). In contrast, for microsatellites we found no significant genetic structure within the Atlantic (*F*_ST_ from -0.0026 to 0.0025, *P* > 0.05; *D*_est_ from 0.0000 to 0.0017) nor within the Mediterranean (*F*_ST_ = -0.0003, *P* > 0.05; *D*_est_ = 0.0000, *P* > 0.05; Table 3). Power analyses indicated that the minimum amount of genetic divergence detectable from our data is a *F*_ST_ = 0.0028 with a ≥ 95% confidence. The PCA results (Fig 3b) show the existence of two groups split along the PC1, which explains most of the variation (PC1: *F*_ST_ = 0.0116, 68% of total inertia, *P* < 0.001). No significant separation along the PC2 was found (PC2: *F*_ST_ = 0.0019, 11% of total inertia, *P* = 1.0000). Also, no evidence of isolation-by-distance within the Atlantic was found (*a* = 0.0028, *b* = 2.1 × 10^−6^; *P* > 0.05). The best AMOVA grouping found was: (MAR1, ROC, MAR2, MAR3) (MED1, MED2), and as the other analyses suggests the separation of the Atlantic and Mediterranean populations (Table E in S1 Appendix). Geneland analyses revealed two clusters corresponding to the Atlantic and Mediterranean populations. The two independent runs performed with 9 and 7 loci, showed the same pattern with two clusters identified (Fig 4).

### Demographic analyses

For mtDNA COI demographic histories were inferred for MAR1/ROC, MAR2/MAR3 and MED populations independently. No deviations from the neutrality was found using the Tajima’s *D* for most populations analysed or Fu’s *F*_S_ (*P* > 0.05, Table G in S1 Appendix), except for MAR1/ROC (*F*_S_ = -2.945, *P* < 0.01) suggesting population expansion. Small Rozas’s *R*_2_ values were found for MAR1/ROC and MED (0.057 and 0.067, respectively), whereas a higher *R*_2_ was resulted for MAR2/MAR3 (0.119).

For ATL and MED microsatellite data, no bottlenecks were detected using the Wilcoxon tests for heterozygosity excess (*P* > 0.05). However the Wilcoxon tests for heterozygosity deficiency was significant for the two regions, suggesting population expansion (Table H in S1 Appendix).

In the DIYABC analysis, scenario 4 (both ATL and MED population sizes change after population split) was the best supported with higher posterior probability (mean *P* = 0.86, 95% CI = 0.74–0.89; Table 4; Fig A in S1 Appendix) relatively to other scenarios (between *P* = 0.00 and 0.19; Table 4; Fig A in S1 Appendix). Based on the logistic estimate, the Type error I estimated for scenario 4 was 4.6 and the Type error II was 0.23. Under scenario 4, the effective population size estimation of both ATL (*N*_ATL_) and MED (*N*_MED_) did not produce distinct modes regardless the large priors given. All other estimated parameters showed distinct modes (Fig C in S1 Appendix). The simulations showed that both populations’ sizes after divergence (*Nb*_ATL_ and *Nb*_MED_) were large (Table 4; Fig C in S1 Appendix) with modal values of 5370 (95% CI: 1410–36200) and 7480 (95% CI: 1760–37500), respectively. Time since divergence was estimated in 6690 generations (95% CI: 2210–45100) for scenario 4, which dates the separation between ATL and MED populations around 60000 years before present (yBP; 95% CI: 19890–405900). The posterior distributions of the mutation rates suggested that the prior range selected was appropriate for the analysed data set (Fig C in S1 Appendix). Model check showed that the observed and the simulated data were similar (Fig C in S1 Appendix), suggesting a good model fit.

**Table 4:** Posterior parameters values for each scenario estimated using DIYABC.

Scenario	Parameter	Mean	Median	Mode	95% CI		*P* (95% CI)
					lower	upper	
S1	*N*_ATL_	8.92 x 10^4^	6.92 x 10^4^	5.37 x 10^4^	1.43 x 10^4^	2.77 x 10^5^	0.00 (0.00–0.01)
	*N*_MED_	1.85 x 10^5^	1.63 x 10^5^	1.04 x 10^5^	3.24 x 10^4^	4.52 x 10^5^	
	*t*	9.48 x 10^3^	5.39 x 10^3^	2.40 x 10^3^	6.40 x 10^2^	4.60 x 10^4^	
S2	*N*_ATL_	6.79 x 10^4^	5.66 x 10^4^	3.85 x 10^4^	1.63 x 10^4^	1.80 x 10^5^	0.00 (0.00–0.01)
	*N*_MED_	3.37 x 10^5^	3.55 x 10^5^	4.97 x 10^5^	8.74 x 10^4^	4.96 x 10^5^	
	*t*	1.52 x 10^4^	1.11 x 10^4^	4.83 x 10^3^	2.05 x 10^3^	5.45 x 10^4^	
	*t1*	7.10 x 10^3^	5.70 x 10^3^	9.74 x 10^2^	3.60 x 10^2^	1.87 x 10^4^	
	*Nb*_MED_	1.59 x 10^4^	1.44 x 10^4^	9.27 x 10^3^	1.43 x 10^3^	3.72 x 10^4^	
S3	*N*_ATL_	9.97 x 10^5^	9.76 x 10^5^	2.66 x 10^5^	9.23 x 10^4^	1.96 x 10^6^	0.14 (0.11–0.26)
	*N*_MED_	1.65 x 10^5^	1.39 x 10^5^	1.01 x 10^5^	2.84 x 10^4^	4.40 x 10^5^	
	*t*	1.37 x 10^4^	1.10 x 10^4^	6.22 x 10^3^	2.13 x 10^3^	4.36 x 10^4^	
	*t1*	1.08 x 10^4^	1.06 x 10^4^	7.87 x 10^3^	1.66 x 10^3^	1.96 x 10^4^	
	*Nb*_ATL_	1.08 x 10^4^	7.89 x 10^3^	2.51 x 10^3^	6.88 x 10^2^	3.49 x 10^4^	
**S4**	*N*_ATL_	1.18 x 10^6^	1.23 x 10^6^	1.98 x 10^6^	1.74 x 10^5^	1.98 x 10^6^	**0.86 (0.74–0.89)**
	*N*_MED_	2.70 x 10^5^	2.68 x 10^5^	1.98 x 10^5^	5.12 x 10^4^	4.89 x 10^5^	
	*t*	1.42 x 10^4^	1.12 x 10^4^	6.69 x 10^3^	2.21 x 10^3^	4.51 x 10^4^	
	*t1*	7.11 x 10^3^	5.65 x 10^3^	2.07 x 10^3^	6.35 x 10^2^	1.89 x 10^4^	
	*Nb*_ATL_	1.38 x 10^4^	1.15 x 10^4^	5.37 x 10^3^	1.41 x 10^3^	3.62 x 10^4^	
	*Nb*_MED_	1.55 x 10^4^	1.34 x 10^4^	7.48 x 10^3^	1.76 x 10^3^	3.75 x 10^4^	

It is presented the mean, median and modal value, the lower and upper 95% credible intervals for each estimated parameter. Also the probability *P* (95% CI) of each scenario under consideration is shown and the best supported scenario is emphasized in bold. Contemporary Atlantic and Mediterranean *N*_e_ (*N*_ATL_ and *N*_MED_), Atlantic and Mediterranean *N*_e_ after divergence (*Nb*_ATL_ and *Nb*_MED_), time since population separation (*t*) and end of time of populations size changes (*t1*). For more details and schematic representation please see Fig A in S1 Appendix. Values are in generation time.

### Analyses of biological data

The length distributions in the three main areas analysed (MAR, ROC and MED) were statistically different (Kruskal-Wallis test, *H* = 78.5, *P* < 0.001), with Mediterranean specimens attaining significantly smaller sizes than in Atlantic localities (Fig B in S1 Appendix).

## Discussion

### Atlantic-Mediterranean genetic barrier

The results obtained for the Mediterranean grenadier suggest the existence of a genetic barrier between Atlantic and Mediterranean populations. This conclusion was sustained by several analyses performed (PCA, AMOVA, Bayesian clustering and *F* statistics) with both nuclear and mitochondrial markers supporting the existence of significant genetic divergence, or a “genetic break”, between the two basins (*F*_ST_ >0.02, *Φ*_ST_ >0.62 and *D*_est_ >0.01). Together with the apparent absence of shared mtDNA haplotypes, this suggests highly restricted or no connectivity between populations on either side of the Strait of Gibraltar. Direct exchange across the Strait of Gibraltar by the active dispersal of the movements of grown fish is improbable given the species’ deep vertical distribution [26, 28, 29, 84] relatively to the shallow strait’s bathymetry. It has been shown recently that the shallow depth of the Strait of Gibraltar has a major effect in restricting dispersal capabilities of other deep-sea species, such as the shark *Centroscymnus coelolepis* [11]. Bathymetry is also a major player in shaping the population connectivity in a close relative of *C*. *mediterraenus*, the roundnose grenadier (*C*. *rupestris*, [7]) and in other fish species such as the bluemouth rockfish (*Helicolenus dactylopterus*, [8]) and tusk (*Brosme brosme*, [9]). While bathymetry may be an obstacle to active fish dispersal, it is less clear how gene flow though the transport of pelagic early-life stages is restricted. However, such gene flow between Atlantic and Mediterranean requires that fertilized eggs and larvae have the ability of rising in the water column reaching depths shallow enough to be transported by currents across Gibraltar sill (284 m deep). Lack of knowledge on depth of occurrence and duration time of this species early life stages difficult any prediction of gene flow patterns. Early ontogeny of *C*. *mediterraneus* and macrourids in general, is poorly known since eggs and larvae have been occasionally identified or collected for only a few species [85–92]. Marshall [93] suggested that grenadiers reproduced near the sea floor and that fertilized eggs float to near the thermocline where they hatch and feed, and subsequently descend to the adult habitats as they grow up. Although at the time this theory was controversial, recent studies [94, 95] using otoliths microchemistry have confirmed this pattern for the species analysed (*C*. *rupestris*, *C*. *acrolepis* and *C*. *marginatus*). Although grenadiers seem to exhibit ontogenetic downward migration, few species have been properly studied and vertical migration distances and timing seem to vary among species [95]. Currently there is no information on the early life stages of *C*. *mediterraneus*, however larvae from *Coryphaenoides sp*. were collected from epipelagic layers (<200 m) from the Aegean Sea (eastern Mediterranean [96, 97]). Since in the Mediterranean only two species of *Coryphaenoides* are reported (*C*. *mediterraeus* and *C*. *guentheri*), and both species share similar depth of occurrence and population genetic structure (this study and Catarino et al. *in prep*.), the early life stages collected at Aegean Sea suggest that *C*. *mediterraneus* larvae may indeed reach the epipelagic layer. However oceanographic processes, such as upwelling or eddies may also have some role in the transportation of this early life stages to such shallow layers, since their abundance at collections sites was low [96, 97]. Even if *C*. *mediterraneus* early life stages can reach layers shallow enough to cross the Strait of Gibraltar, those egg/ larvae have to be viable and adapt to the dramatic differences in the environmental conditions (temperature and salinity) between Mediterranean and Atlantic. The higher temperatures in the Mediterranean (e.g. [44]) may also accelerate egg incubation time and larval development, shortening the period of the pelagic life stage and the dispersion time [85]. The lack of shared haplotypes for *C*. *mediterraneus* COI sequences between Atlantic and Mediterranean samples (see Fig 2) supports the hypothesis of an absence of gene exchange between the two basins.

For species with planktonic stages, the AOF [19] has been considered as an important genetic barrier [98–104], and its effect compared to the Strait of Gibraltar is still under discussion (reviewed by [12]). Although our sampling did not include samples from Alboran Sea, it is more likely that the Strait of Gibraltar rather than AOF plays a more important role on the genetic pattern found for *C*. *mediterraneus* Mediterranean population. The AOF influences only the top 300 meters depth [19], and even if the eggs and larvae of this species get partly retained at surface layers, below that depth the movements of plankton and fish should not be affected. From this perspective, the 1000 m deep dwelling Mediterranean grenadier juveniles and adults should be able to freely interact beneath the front without any restrictions between the Alboran Sea and the western Mediterranean basin, as has been reported for other deep-sea species [105].

Specimens from the Mediterranean attain a smaller average size than those collected in the Atlantic (Fig B in S1 Appendix). These findings are in accordance with a previous study conducted for *C*. *mediterraneus* [36] and reinforce the idea of a segregated Mediterranean population. This pattern seems to be a common trend for several other Mediterranean fishes [11, 36, 106], where a combination of factors such as temperature and food availably may have an important role (see discussion in Massutí et al [36] and Catarino et al [11]).

The estimated *p*-distance values between the two *C*. *mediterraneus* haplo-groups found (Atlantic and Mediterranean) are much lower (0.54% ± 0.20%) than those found between *C*. *mediterraneus* and other closely related *Coryphaenoides* species analysed (from 5.74% ± 0.95% to 11.29% ± 1.31%). According to the phylogenetic reconstruction using mtDNA COI region [61] the closest living relative to *C*. *mediterraneus* is *C*. *striaturus*. The estimated *p*-distance values between these two species are about one order of magnitude higher than those reported herein between the Atlantic and Mediterranean populations of *C*. *mediterraneus*. This suggests that the Atlantic and Mediterranean populations of *C*. *mediterraneus* should be treated as belonging to the same species. However, the fact that the Mediterranean population appears isolated, together with the size differences (smaller maximum size, and consequently smaller first maturity size), suggest that this population may be on its separated evolutionary path.

### Genetic pattern within the Mediterranean

Many studies had reported the West–East transition as one of the major biogeographic discontinuities within the Mediterranean Sea for diverse taxa, such as for sea-grass, prawns and fish [14–17]. In contrast, we found no significant genetic difference of *C*. *mediterraneus* from the Western and Eastern Mediterranean (both types of markers after FDR correction). This finding may be due to the fact that the Strait of Sicily is about 100 meters deeper that the Strait of Gibraltar which may be enough for the *C*. *mediterraneus* early life stages easily overpasses this potential barrier. Furthermore, local strong and periodical upwelling processes, (e.g. [18]), bring deep-water to surface layers which may also facilitate the transport of egg/larvae between the two Mediterranean basins [107]. The exchange of only a limited number of migrant across the Strait of Sicily and/or Messina may be enough to prevent genetic differentiation between the Western and Eastern Mediterranean populations. Other deep-sea species such as the shrimp *Aristeus antennatus*, exhibit a similar genetic pattern to that found in our studied species, also suggesting that the Strait of Sicily and/or Messina has little or no influence restricting genetic connectivity between west and east basin in deep-sea species with pelagic stages [108].

The lack of genetic differentiation between West and Eastern Mediterranean basins should not be the result of a poor sampling design, since the results from the power analyses indicated that our sampling design should be able to detect genetic divergence as low as *Φ*_ST_ = 0.0643 for mtDNA and *F*_ST_ = 0.0028 for microsatellites.

### Genetic pattern within Atlantic

While the general genetic pattern across the Atlantic-Mediterranean transition and within the Mediterranean is quite simple, a more complex genetic pattern was found within the Atlantic. Microsatellite markers displayed no genetic structure, suggesting a single panmictic population within the Atlantic. The lack of genetic heterogeneity found with the nuclear markers is in accordance with other studies for the same area performed with other *Coryphaenoides* species, such as *C*. *armatus* and *C*. *brevibarbis* [109, 110], suggesting that the CGFZ, the Sub-Polar Front and the topographic structure of the Mid-Atlantic Ridge have little effect on the genetic connectivity in this deep-sea species.

However, for the mtDNA COI, genetic differentiation was found across the CGFZ (between MAR1/ROC and MAR2/MAR3 areas), with a marked higher level of genetic variability at the southern areas (Table 2). The results showed no signals of recent population contractions that could justify such a low genetic diversity in the northern areas, however it is feasible that the tests employed could miss an older bottleneck event [111]. Contrasting patterns of population structure between mitochondrial and nuclear markers are often ascribed to sex-biased dispersal. Since for nuclear markers no genetic structure was found, this would be indicative of male-mediated gene flow, while females would exhibit philopatric behaviour. Although female philopatry has been described in deep-sea sharks [112] such behaviour was never described for any macrourid species and it seems unlikely given the species’s life cycle. The most likely explanation for the contrasting genetic pattern found with the mtDNA and nuclear markers is due the lower effective population size of the mtDNA compared to the nuclear genome, which turn it more susceptible to stochastic events [113].

### Demographic history and population divergence

No evidences of past population contractions for *C*. *mediterraneus* populations were found with the tests employed both for mtDNA and microsatellites. The best supported scenario chosen by DIYABC showed elevated modal values of *N*_e_ after the Atlantic—Mediterranean separation (*Nb*_ATL_ and *Nb*_MED_, Table 4; Fig C in S1 Appendix) suggesting that no severe reductions in the populations has occurred. According to the ABC results the separation between Atlantic and Mediterranean populations (60210 yBP, 95% CI: 19890–405900 yBP) is likely to have occurred during the last glacial period at the late Pleistocene (110000–12000 yBP [114]). This estimation is calculated using an assumed generation time of nine years [30], however further studies are needed in order to produce a more accurate generation length estimate. The relatively wide credible intervals estimated for the split suggest that an earlier separation cannot be entirely ruled out, however the separation occurred no later than the last glacial period. The estimated splitting period between the Atlantic/Mediterranean *C*. *mediterraneus* is similar to the one provided for another deep-sea species for the same region (*C*. *coelolepis* and *Chimaera monstrosa*, [11, Catarino et al. *in prep*.]. Furthermore, the last glacial period has been earlier suggested as particular severe for deep-sea species where the contact with the Mediterranean was thought to be lost or very limited [115].

In summary, the Atlantic and Mediterranean populations of *C*. *mediterraneus* are genetically distinct due to the limited genetic exchange across the Strait of Gibraltar. This physical barrier prevents the direct interactions of adult fish from the two basins due its shallow bathymetry and even the pelagic early life stages of this species seems not be able overpass this shallow barrier.

## Supporting information

S1 Appendix A file containing all tables and figures in supporting information. (PDF)

S2 Appendix *Coryphaenoides mediterraneus* alignment haplotypes. This file contains all 10 haplotypes found for mtDNA COI in the studied areas for *C*. *mediterraneus* (Cmed). The file is in fasta format. Haplotypes frequencies: H1-15; H2-108; H3-4; H4-1: H5-2; H6-1; H7-14; H8-49; H9-1; H10-2.
(FST)

S3 Appendix *Coryphaenoides mediterraneus* microsatellites genotypes. This file contains the microsatellites genotypes for the 375 specimens of *C*. *mediterraneus* (Cmed). The file is in genepop 3 digits format, which is widely used and can be easily converted in other types of files. It contains the genotypes for the 9 nuclear loci screened in the 8 localities studied: MAR1- Northern Mid-Atlantic Ridge; MAR2- Middle Mid-Atlantic Ridge; MAR3- Southern Mid-Atlantic Ridge; ROC- Rockall; MED1- Western Mediterranean; MED2- Eastern Mediterranean.
(TXT)
