# Supplementary material for: The role of the Strait of Gibraltar in shaping the genetic structure of the Mediterranean Grenadier, Coryphaenoides mediterraneus, between the Atlantic and Mediterranean Sea
Source: PLoS One. 2017 May 1;12(5):e0174988. doi: 10.1371/journal.pone.0174988 (PMC5411037; doi:10.1371/journal.pone.0174988)
Supplement: S1 Appendix — (PDF) [file pone.0174988.s001.pdf]

S1 Appendix

Diana Catarino, Sergio Stefanni, Per Erik Jorde, Gui M. Menezes, Joan B. Company, Francis Neat and Halvor Knutsen 2017. The role of the Strait of Gibraltar in shaping the genetic structure of the Mediterranean grenadier, *Coryphaenoides mediterraneus*, between the Atlantic and Mediterranean Sea. PlosOne.

Table A. Preliminary analyses of pairwise F_{ST} values between sampling sites within Western (W1, W2 and W3) and Eastern (E1 and E2) Mediterranean. Based on the lack of genetic heterogeneity, all Western localities were grouped in one Western Mediterranean site (MED1) and the Eastern localities in one Eastern Mediterranean site (MED2) for all the analyses in the main manuscript. For all, $P > 0.05$ for the exact G test of allele frequency heterogeneity over loci. Same order as in Table 1.

	**W1MED**	**W2MED**	**W3MED**	**E1MED**	**E2MED**
**W1MED**	—				
**W2MED**	-0.0090	—			
**W3MED**	-0.0049	-0.0155	—		
**E1MED**	0.0018	-0.0050	-0.0096	—	
**E2MED**	-0.0091	-0.0098	0.0130	0.0023	—

Table B. Nuclear microsatellite loci combined in multiplexes (Set) and annealing temperature (T_a). Black lines separating the loci refer to how the loci were run (as multiplex or solo) during the PCR and the "Set" refers to the loci arrangement for the ABI 3130*xl* run. Estimated quantities: H_T, total heterozygosity; A, number of alleles scored per locus; R_S, mean allelic richness (minimum sample size of 95 individuals); F_{IS} deviations from Hardy-Weinberg equilibrium; F_{ST}, level of genetic differentiation among all sampled locations; overall[1] for nine loci; overall[2] for 7 loci (removing Crup7 and CaraA10). Significant at alpha = *0.05, ** 0.01, *** 0.001, after FDR approach.

Loci	Set	T_a (°C)	H_T	A	R_s	F_{IS}	F_{ST}
**Crup7**	**1**	56	0.926	35	28.1	**0.1094*** **	**0.0269*** **
**CaraA106a**	**1**	56	0.475	18	13.2	-0.0232	**0.0390*** **
**Mbe03**			0.245	6	4.4	-0.0116	**0.0110***
**CaraA109**	**2**	50	0.671	13	9.8	-0.0076	0.0001
**CaraC7**	**3**	58	0.468	12	8.8	-0.0407	**0.0134*** **
**CaraA10**	**4**	56	0.164	5	2.8	**0.2356** **	0.0036
**CaraA102**			0.021	2	1.9	-0.0105	0.0036
**CaraB1**			0.044	4	3.0	0.1150	**0.0117** **
**CaraC1**			0.789	12	9.6	-0.0059	**0.0028***
**Overall[1]**			0.422			**0.0263*** **	**0.0149*** **
**Overall[2]**			0.387			-0.0143	**0.0116*** **

Table C. DIYABC summary statistics. Fields with an observed value correspond to the statistics used to build the reference table, the model choice analyses and to estimate the parameters. The observed value corresponds to the estimation in the real dataset. For each scenario the x indicates a summary statistic where the simulated value is similar to the observed value; the x followed by a *, **or *** indicates summary statistics where observed and simulated data were significantly different at 0.05, 0.01 or 0.001, respectively. Summary statistics not used in the reference table construction (i.e. statistics with no observed value calculated) were then used for model checking analysis.

	Summary statistics	Observed value		Scenario 1		Scenario 2		Scenario 3		Scenario 4	
		ATL	MED	ATL	MED	ATL	MED	ATL	MED	ATL	MED
	**One Sample**										
Microsats	Mean number of alleles	8.8889	7.1111	x**	x*	x**	x*	x	x*	x	x
	Mean genetic diversity	0.4423	0.3809	x**	x**	x**	x*	x*	x*	x*	x*
	Mean size variance										
	Mean Garza-Williamson's M	0.5063	0.6095	x***	x*	x***	x	x***	x***	x***	x**
mtDNA COI	Number of haplotypes	4.0000	4.0000	x*	x	x*	x	x	x	x	x
	Number of segregation sites	3.0000	5.0000	x*	x	x*	x	x	x	x	x
	Mean of pairwise differences										
	Variance of pairwise differences										
	Tajima's D	0.1190	-1.1593	x	x	x	x	x*	x	x*	x
	Private segregating sites										
	Mean of numbers of the rarest nucleotide at segregating sites	7.3333	4.0000	x	x	x	x	x	x	x	x
	Variance of numbers of the rarest nucleotide at segregating sites										
	**Two Sample**										
Microsats	Mean number of alleles	9.4444		x**		x**		x*		x*	
	Mean genetic diversity	0.4170		x***		x***		x**		x*	
	Mean size variance										
	Fst	0.0343		x		x		x		x	
	Classification index	0.8736	0.8115	x***	x***	x***	x***	x*	x**	x**	x**
	Shared allele distance										
	$(d\mu)^2$ distance										
mtDNA COI	Number of haplotypes	8.0000		x		x		x		x	
	Number of segregation sites										
	Mean of pairwise differences (W)	0.5866		x*		x		x		x	
	Mean of pairwise differences (B)										
	Fst (Hudson *et al.*, 1992)	0.6670		x*		x		x		x	

Table D. Average genetic *p*-distances based on mtDNA COI for *C. mediterraneus* populations and other *Coryphaenoides* species. Average *p*-distances are shown below the diagonal and standard error estimates are shown above the diagonal. The analysis involved 19 nucleotide sequences, each with 598 bp. Abbreviations: Cstr, *C. striaturus*; Cmur, *C. murrayi*; Ccar, *C. carapinus*; Cbre, *C. brevibarbis*, Cmed, *C. mediterraneus*; ATL, Atlantic; MED, Mediterranean.

	Cstr	Cmur	Ccar	Cbre	Cmed ATL	Cmed MED
**Cstr**	–	0.013	0.013	0.013	0.010	0.010
**Cmur**	0.115	–	0.013	0.013	0.012	0.012
**Ccar**	0.109	0.112	–	0.012	0.013	0.013
**Cbre**	0.110	0.120	0.105	–	0.013	0.013
**Cmed ATL**	0.057	0.092	0.106	0.112	–	0.002
**Cmed MED**	0.060	0.095	0.108	0.113	0.005	–

Table E. Hierarchical AMOVA analyses for the mtDNA COI fragment and the 9 microsatellite loci of the Mediterranean grenadier. Seven grouping criteria were tested and the highest support is found for those displaying the largest F_{CT} in relation to F_{SC}. In this perspective the best grouping option is shown in green for mtDNA and in orange for microsatellite markers. Sampled sites abbreviations are given in Table 1.

Grouping	Source of variation	mtDNA COI			Microsatellites		
		**% of variance**	**Φ statistics**	***P***	**% of variance**	**Φ statistics**	***P***
**Global panmixia** - total data set	Among populations	62.8	Φ_{ST}= 0.6279	**<0.001**	1.3	F_{ST}= 0.0134	**<0.001**
	Within populations	37.2			98.7		
**Two regions - Atlantic vs. Mediterranean**	Among groups	70	Φ_{CT}= 0.6999	**<0.05**	2.4	F_{CT}= 0.0239	**<0.001**
(MAR1, MAR2, MAR3, ROC) (MED1, MED2)	Among populations within groups	4.2	Φ_{SC}= 0.1392	**<0.001**	0.1	F_{SC}= 0.0013	0.221
	Within populations	25.8	Φ_{ST}= 0.7417	**<0.001**	97.5	F_{ST}= 0.0252	**<0.001**
**Three regions**	Among groups	61.1	Φ_{CT}= 0.6108	0.0506	1.4	F_{CT}= 0.0141	**0.003**
(MAR1, MAR2, MAR3) (ROC) (MED1, MED2)	Among populations within groups	5.8	Φ_{SC}= 0.1491	**<0.001**	0.1	F_{SC}= 0.0014	0.312
	Within populations	33.1	Φ_{ST}= 0.6689	**<0.001**	98.5	F_{ST}= 0.0154	**<0.001**
(MAR1, ROC) (MAR2, MAR3) (MED1, MED2)	Among groups	66.3	Φ_{CT}= 0.6628	**<0.01**	1.6	F_{CT}= 0.0160	**<0.001**
	Among populations within groups	1	Φ_{SC}= 0.0299	0.1460	0.05	F_{SC}= 0.0005	0.4337
	Within populations	32.7	Φ_{ST}= 0.6728	**<0.001**	98.4	F_{ST}= 0.0165	**<0.001**
(MAR1, ROC, MAR2) (MAR3) (MED1, MED2)	Among groups	65	Φ_{CT}= 0.6500	**<0.05**	1.7	F_{CT}= 0.0172	**<0.001**
	Among populations within groups	2.9	Φ_{SC}= 0.6786	**<0.01**	0	F_{SC}= 0.0000	0.4915
	Within populations	32.1	Φ_{ST}= 0.0818	**<0.001**	98.3	F_{ST}= 0.0172	**<0.001**

Table F. Pairwise F_{ST} calculated using Arlequin.

	MAR1	MAR2	MAR3	ROC	MED1	MED2
MAR1	–					
MAR2	-0.0012	–				
MAR3	0.0026	-0.0023	–			
ROC	0.0010	-0.0024	0.0017	–		
MED1	**0.0199*** **	**0.0426*** **	**0.0312*** **	**0.0218*** **	–	
MED2	**0.0260*** **	**0.0449** **	**0.0371*** **	**0.0264*** **	0.0011	–

Table G. Neutrality test for *C. mediterraneus* mtDNA COI sequences. * $P < 0.01$.

	**MAR1/ROC**	**MAR2/MAR3**	**Mediterranean**
Number of individuals	79	48	66
Tajima's *D*	-1.311	0.119	-1.159
Fu's F_S	**-2.945***	-0.019	-0.559
Rozas's R_2	0.057	0.119	0.067

Table H. Bottleneck tests results for *C. mediterraneus* samples. P refers to the probability of each test. Different combinations of percentage of stepwise mutations and variance were tested in TPM model: **1**) 70, 10; **2)** 70, 30; **3)** 90, 10; **4)** 90, 30, for the percentage and variance, respectively.

	**P (H deficient)**				
**Population**	**SMM**	**TPM**			
		**1**	**2**	**3**	**4**
**Atlantic**	0.00098	0.00098	0.00098	0.00098	0.00098
**Mediterranean**	0.00098	0.00488	0.00684	0.00195	0.00195

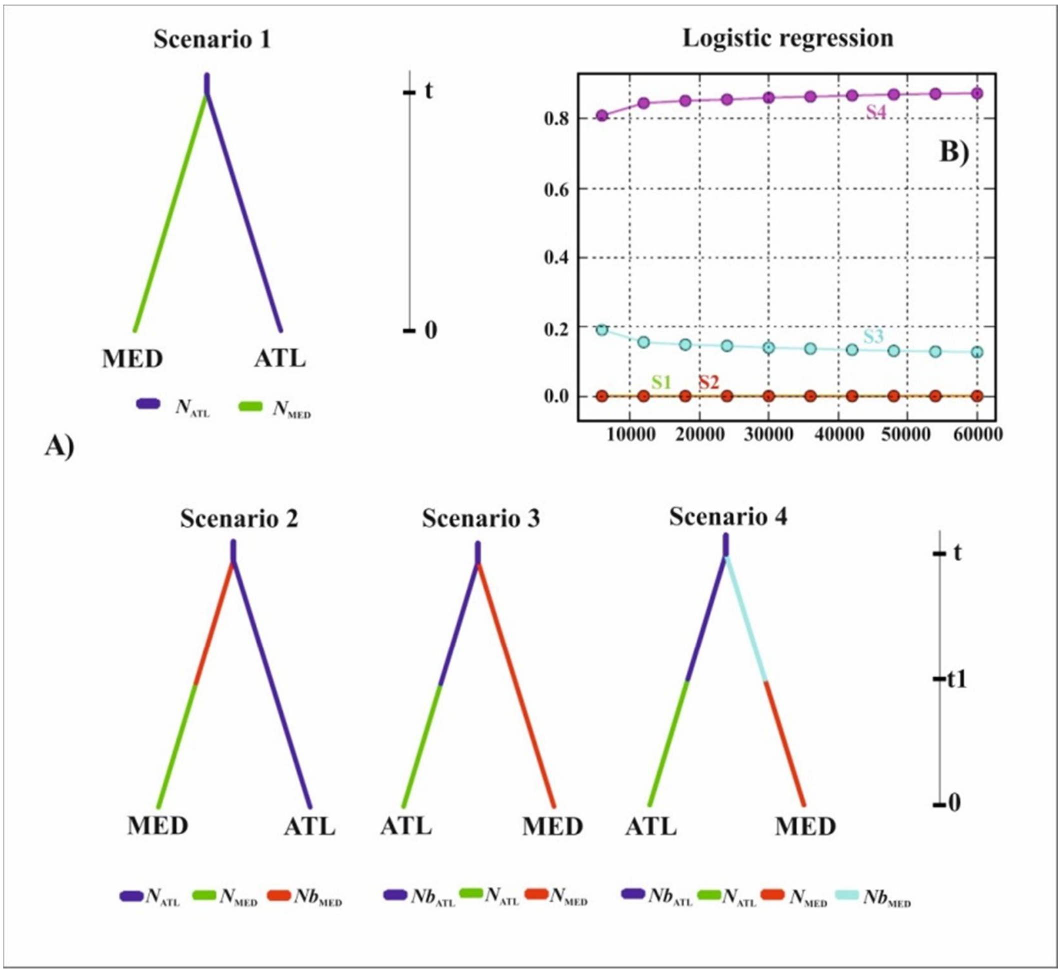

Figure A. DIYABC scenarios under consideration for *C. mediterraneus* populations. **A)** schematic representation of the four alternative scenarios used for Approximate Bayesian computation simulations. All scenarios assume that at present time $t(0)$, there are two populations Atlantic (ATL) and Mediterranean (MED) and that they diverged from a single population in the past at t generations. Nb_{ATL} and Nb_{MED} indicate periods where population size was allowed to change. For detailed information on each scenario see Material and Methods section; **B)** Relative posterior probability of the demographic scenarios estimated using the logistic regression approach. Scenario 1 and 2 are overlapping at the bottom of the graph (see Table 4 for probability values and 95% CI).

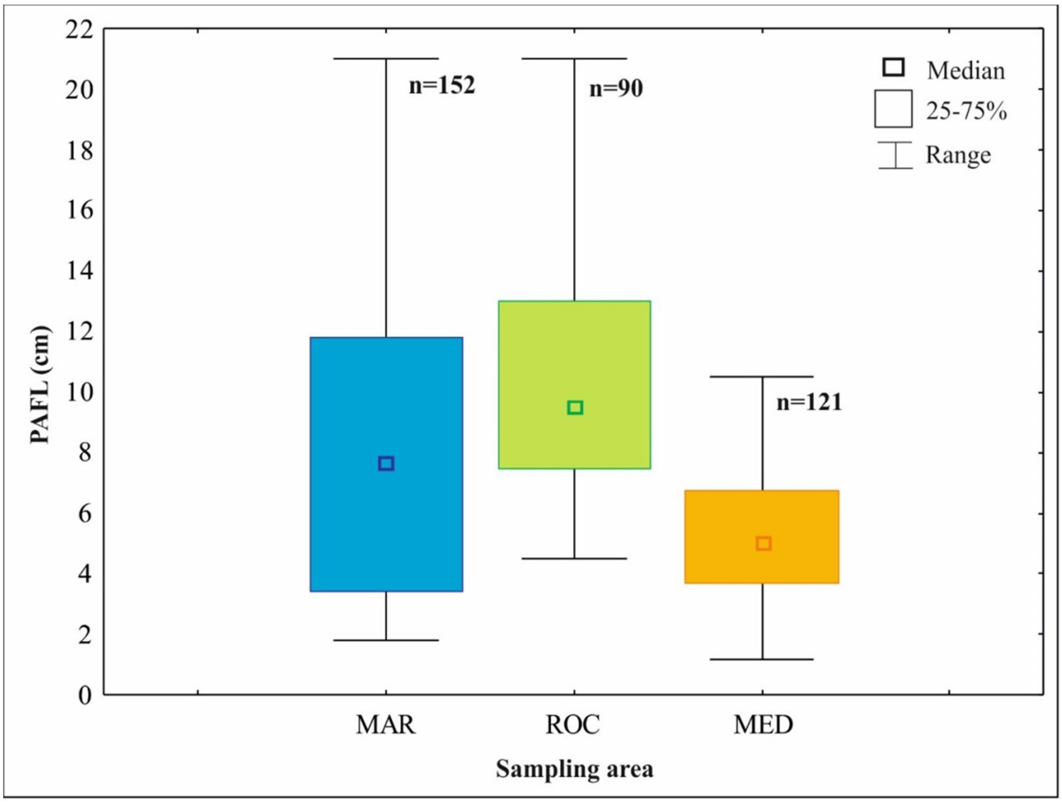

Figure B. Resume of the length data based on the indicated number (*n*) of specimens collected in each of the main studied areas. Length was recorded as the pre-anal fin length (PAFL). Sampling area abbreviations are given in Table 1.

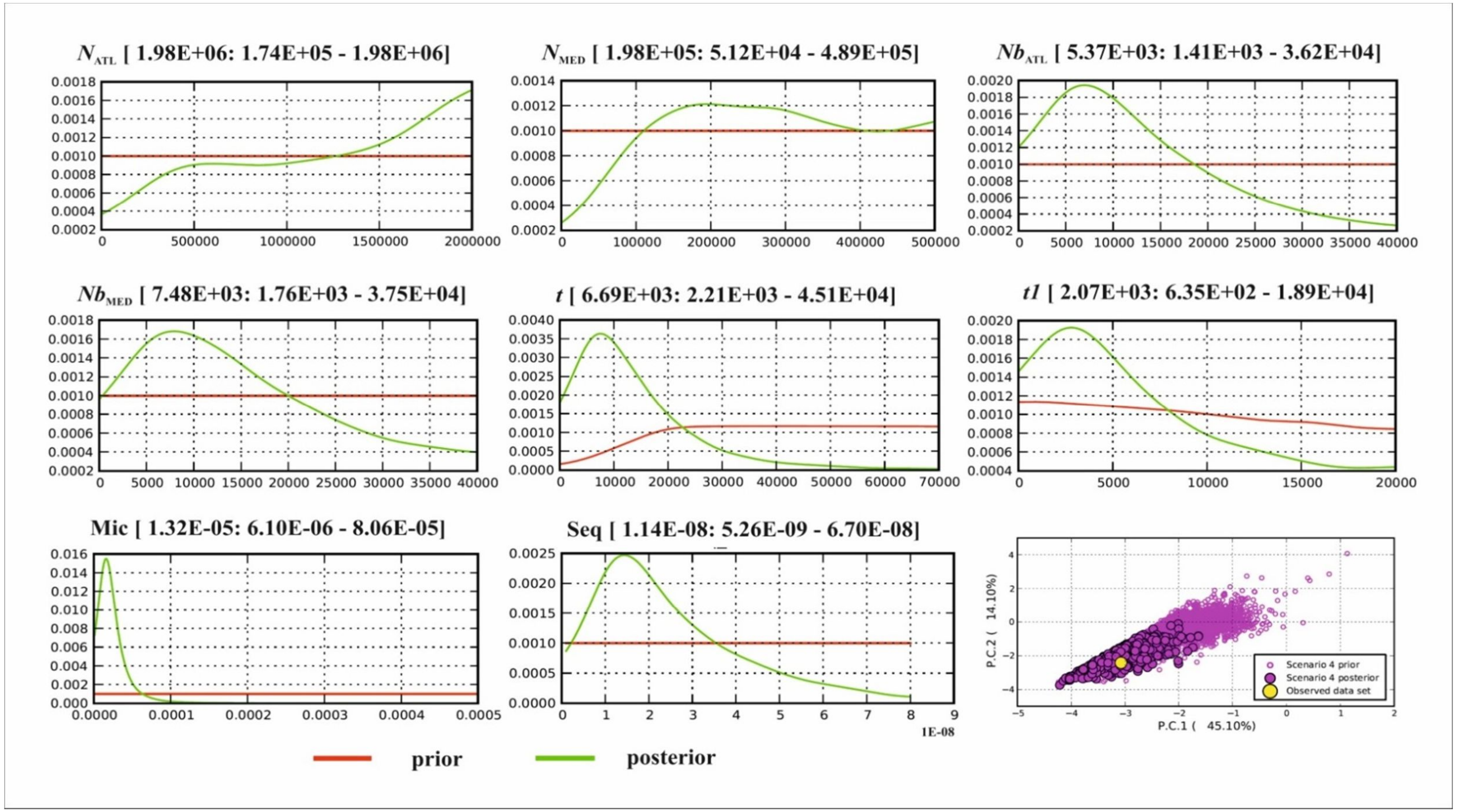

Figure C. DIYABC posterior probability distributions (green lines) for each parameter estimated under scenario 4. Besides the distributions it is also shown the modal values (95% Credible Interval) for Atlantic and Mediterranean N_e (N_{ATL} and N_{MED}), Atlantic and Mediterranean N_e after divergence (Nb_{ATL} and Nb_{MED}), time since divergence (t), $t1$ and the distributions for the mutation rates for microsatellites (Mic) and mtDNA (Seq). The last image refer to the "model checking" analyses using a PCA to evaluate the model fitting of the dataset, using a different set of summary statistics from those used to estimate the parameters.
